# The case of pulmonary tuberculosis with COVID-19 in an Indian male-a first of its type case ever reported from South Asia

**DOI:** 10.11604/pamj.2020.36.374.24260

**Published:** 2020-08-31

**Authors:** Sankalp Yadav, Gautam Rawal

**Affiliations:** 1 Department of Medicine and Tuberculosis, Shri Madan Lal Khurana Chest Clinic, Moti Nagar, NDMC, New Delhi, India,; 2 Department of Respiratory Intensive Care, Max Super Specialty Hospital, Saket, New Delhi, India

**Keywords:** Tuberculosis, COVID-19, SARS-CoV-2

## Abstract

We report the first case of tuberculosis with COVID-19 from South Asia. The patient was a 43 years old Indian male. He reported to us in the outpatient department with chief complaints of cough with expectoration associated with fever, chest pain, and night sweats. The diagnosis of tuberculosis in the current pandemic of COVID-19 required a high degree of suspicion to rule out the SARS-CoV-2 infection along with the infection of Mycobacterium tuberculosis. The clinical presentations in the two diseases are quite similar and thus the present case will serve as a tool to help the clinicians handling cases of both the viral and bacterial infection across the globe.

## Introduction

The world is currently paralyzed by the pandemic of COVID-19 [1]. This infectious disease has resulted in so much morbidity and mortality across the globe [1]. This zoonotic disease which is the mainstay of this pandemic is caused by a group of β - coronavirus named severe acute respiratory syndrome coronavirus 2 (SARS-CoV-2) which is a novel strain of single-stranded RNA viruses [1, 2]. This was first reported from the Wuhan city of China´s Hubei province following a rise in the cases of pneumonia with no obvious reasons in the month of December 2019 [2, 3]. Since then the diseases have spread across the world affecting almost all sub-populations [4, 5]. Tuberculosis (TB) is a bacterial infection known to mankind for a long time [6]. The disease is a major public health problem especially in the low-income countries of Asia, Africa, and Europe [7]. The disease is caused by *Mycobacterium tuberculosis* [7]. For long the management of TB involved a long drug regimen associated with high pill-burden, multiple adverse drug reactions, etc. [8]. The disease becomes even more deadly when the bacterial strain is of drug-resistant type [7]. In the present pandemic where the viral infection is looming large on the communities, the diagnosis, management, and cure of TB is a challenging task. The same becomes even more challenging when the TB is associated with other diseases like COVID-19. In the present paper, the authors present a case of an Indian male diagnosed with TB and COVID-19. To the best knowledge of authors, no such peer-reviewed case has ever been reported from South Asia.

## Patient and observation

A 43-year-old non-diabetic Indian male reported to our outpatient department with chief complaints of cough with expectoration, chest pain, reduced appetite, fever with chills, and night sweats for two weeks. He also complained of breathlessness on exertion and had two episodes of blood in his sputum. The patient explained that the cough was continuous and was relieved after taking cough syrup. He also mentioned that the episodes of fever were initially intermittent and then daily for the last two weeks and were relieved after taking Paracetamol. The chest pain was localized to the middle of the chest and was aggravated on exertion. He was a businessman by profession with no history of smoking, alcoholism, or any other substance abuse. Also, there was no history of any contact of TB or COVID-19 in the family or close contacts. And there was no history of foreign travel in the recent past. But he had reported having traveled by a domestic airline about twenty days back. There was no history of weight loss or any other major illness in the past. On examination his vitals were- pulse-108/minute, arterial BP-130/80 mm of Hg, respiratory rate of 30 breaths/minute, Sp02-89% on room air, temperature- 101-degree centigrade. His Sp02 fell by 70% on room air after waking. On auscultation, there was crepitation on the bilateral middle lobes of the lungs. Also, dyspnea on exertion was noted. The rest of the systemic examination was within normal limits. Considering this as a probable case of TB with COVID-19 he was advised a chest radiograph with sputum microscopy (Ziehl Neelsen (ZN) staining for acid-fast bacilli), Cartridge-based nucleic acid amplification test (CBNAAT) of the sputum and other routine investigations. To check for the COVID-19 he was advised qualitative polymerase chain reaction (PCR) test from the oropharyngeal swab. The results were surprising with *Mycobacterium tuberculosis* detected on sputum fluorescent microscopy and were also confirmed by the CBNAAT. However, there was no resistance to Rifampicin. The results of the PCR were positive for RNA specific to SARS-CoV-2. Besides, the chest radiograph PA-view was suggestive of bilateral consolidations on the middle lobes of lungs with ill-defined borders. The other investigations revealed a low lymphocyte count (1x 10^9^/L) and increased levels of C-reactive protein (CRP) (57 mg/L), lactate dehydrogenase (LDH) (580 U/L), and erythrocyte sedimentation rate (ESR) (70 mm in the 1^st^ hour). Also, a sample for liquid culture (MGIT BACTEC) was sent to the Intermediate Reference Laboratory (IRL) which revealed the growth of *Mycobacterium tuberculosis*. Computed tomography was not performed as the diagnosis was established by other cheaper and faster methods and also the patient was unwilling for the same. All the other routine investigations were within normal limits. He was referred to the nearest designated COVID-19 management center, where he was managed as per national guidelines. Besides, he was also started on an antitubercular treatment of four drugs as per the National Tuberculosis Elimination Program (NTEP) guidelines. He was advised follow-up post completion of his stay at the designated COVID-19 center, but he has not yet reported back for follow-up. Written informed consent was obtained from the patient for using clinical data and images for publication in this study.

## Discussion

The global spread of COVID-19 has overwhelmed the healthcare systems across all the countries [9]. The rapidly progressing viral infection has resulted in a pandemic which has resulted in large scale morbidity and mortality [9]. The spread of COVID-19 in countries that have a high burden of other diseases like TB could have a devastating effect on already crumbling health facilities in these low- and middle-income countries [10]. Also, as reported in the past such pandemics can result in a tendency to overlook other endemic diseases, such as TB [11]. The disease like TB is of a common occurrence especially in the high burden countries [7]. However, the presence of TB with concomitant COVID-19 is very rare and to date, no such peer-reviewed case reports are available in the medical literature from South Asia. Only cases which are not peer-reviewed are available on the internet, and thus cannot be considered as reliable till the peer-review as per the set guidelines is done [12]. The detection of co-infection of TB with SARS-CoV-2 is very important for the early diagnosis, prevention of contact transmission, and proper management of both the infections.

In the past, the co-infection of TB has been reported in the epidemics and pandemics of other viral diseases like SARS, MERS, etc. [12, 13]. Thus, the possibility of TB with COVID-19 should always be considered in high TB burden settings. The first report of TB and COVID-19 co-infection was published by He *et al*. 2020 from China where they reported three cases of the two infections [14]. The present case differs from the three cases of He *et al*. 2020 by his geographic location, no history of TB in the past or in the family and close contacts, no complaints of diarrhea, and no reinfection to date [14]. However, the study by He *et al*. 2020 had limitations of not performing CBNAAT and culture of *Mycobacterium tuberculosis*, on the study subjects [14]. Thus, a diagnosis of drug-resistant TB in their study could not be established. Our patient had drug-sensitive TB and was thus given appropriate treatment as per the drug sensitivity by four antitubercular drugs. All the set guidelines as detailed in the NTEP were followed in our case. The present case showed certain similarities with the study of He *et al*. 2020 in gender, presenting symptoms, laboratory tests which showed low lymphocyte count, higher than normal levels of CRP, LDH, and ESR [14].

The Global Tuberculosis Network (GTN) reported the first-ever global cohort of current or former TB patients (post-TB treatment sequelae) with COVID-19 in eight countries and three continents [12]. This study recruited 49 consecutive patients with an active infection or a history of TB and COVID-19 from 26 centers in Belgium (1), Brazil (Porto Alegre, Rio Grande do Sul State; 1), France (12), Italy (17), Russia (Moscow Region; 6), Singapore (1), Spain (10) and Switzerland (Vaud Canton; 1) [12]. In this study, about 73.5% of the study subjects had pulmonary TB with COVID-19 infection [12]. The mean age was 48 years with male preponderance [12]. About 53.1% of the study subjects were migrants [12]. This study also showed that about 89.6% COVID-19 cases with TB were symptomatic [12] and 85.7% of patients (the majority being drug-sensitive TB) had active TB when they were diagnosed with concomitant COVID-19 [12]. Although with limited knowledge on this topic this study is an important addition to medical literature, but this study has limitations like the omission of latent TB cases, small sample size, and no data on drug-drug interactions, etc. [12]. The concomitant infection of TB and SARS-CoV-2 although rare are seen in patients with poor immunity. In the past reports of the development of TB post acquiring SARS, likely as a result of reactivation of past infection or new infection with *M. tuberculosis*, while temporarily immunosuppressed because of SARS, are available in the literature [15].

## Conclusion

With the paucity of literature, the present case will serve as a valuable source of information for the management of the two simultaneous infections in various countries around the globe. The clinicians should have a high index of suspicion in the present pandemic and should consider evaluating patients for both TB and COVID-19. Besides, larger studies from various centers around the globe are imperative for formulating the new management guidelines for the two infections.
